# Common Benign Nerve Sheath Tumor at an Uncommon Location - Gastric Schwannoma

**DOI:** 10.7759/cureus.62569

**Published:** 2024-06-17

**Authors:** Manjesh K A, Nagarjun Nelluri, Ankith R Narra, Sreedhar R Kota, Kishore Abuji

**Affiliations:** 1 General Surgery, Employees' State Insurance Corporation (ESIC) Medical College, Hyderabad, IND

**Keywords:** rare gastric tumor, benign peripheral nerve sheath tumor, mesenchymal tumour, gastrointestinal stromal tumor (gist), gastric schwannoma

## Abstract

Gastric schwannomas are rare types of gastrointestinal mesenchymal tumours that are slow-growing and mostly benign. They are usually asymptomatic. In some cases, nonspecific gastric symptoms, palpable mass, and bleeding can be seen. A definitive diagnosis requires pathological and immunohistochemical examination, and surgical resection offers an excellent prognosis with uncommon recurrence. We present a case of a 62-year-old woman who underwent exploratory laparotomy and wedge resection with preoperative diagnosis as gastrointestinal stromal tumor and postoperatively diagnosed as schwannoma on histopathology

## Introduction

Schwannomas are spindle cell mesenchymal tumours originating from the nerve sheath of Schwann cells [1]. They are rarely seen in the gastrointestinal tract (GI) and are usually slow-growing asymptomatic neoplasms. The most common site of GI schwannoma is the stomach, which represents 0.2% of all gastric tumours and 4% of all benign gastric tumours [2] and is followed by the colon and rectum, whereas small intestinal and oesophageal occurrence is extremely rare [3, 4]. Preoperative diagnosis is a challenging issue as it mimics the features of other GI mesenchymal tumours and thus makes it difficult to differentiate. We present a case of symptomatic gastric schwannoma, which was diagnosed as a gastrointestinal stromal tumor (GIST) preoperatively.

## Case presentation

**Figure 1:**
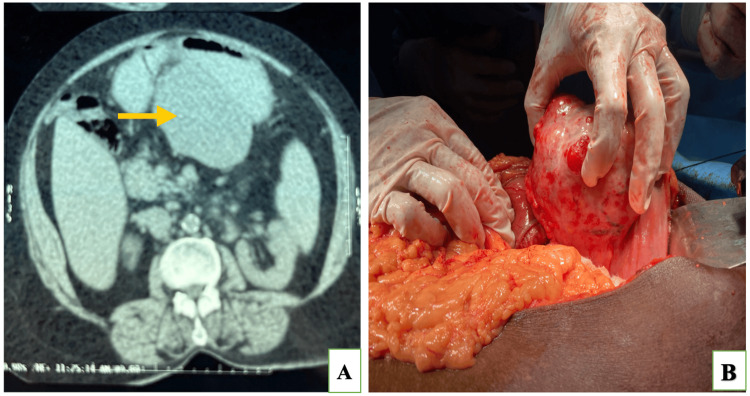
A) Cross-sectional image showing homogenous solid lesion arising from the lesser curvature of the stomach; B) intraoperative image showing solid lesion arising from the lesser curvature of the stomach

A 62-year-old female presented with early satiety and weight loss, for which she underwent an ultrasound of the whole abdomen that revealed a gastric lesion. The patient was admitted to our department for further evaluation of the gastric lesion. The patient complained of early satiety that started one year ago and weight loss of 6 kg in a year without a history of abdominal pain or discomfort. On abdominal examination, it was soft with no tenderness and no palpable mass, and biochemical parameters were within normal limits. On ultrasound, there was a hypoechoic mass lesion in the epigastric region measuring approximately 9.6 ×6.2cm arising from the lesser curvature of the stomach. Upper GI endoscopy revealed submucosal growth with umbilication in the body of the stomach, and contrast-enhanced computed tomography (CECT) of the abdomen showed a well-defined exophytic homogeneously enhancing hypodense soft tissue density mass lesion measuring approximately 9×6.1×8.5cm noted along the lesser curvature of fundus and body of the stomach suggestive of GIST (Figure 1A). After obtaining consent, the patient was prepared for the surgery with a provisional diagnosis as GIST, and on exploratory laparotomy, there was about 10×9.5×6cm lesion arising from the lesser curvature of the stomach and proceeded with excision of the lesion with a 2cm margin (Figure 1B).

**Figure 2:**
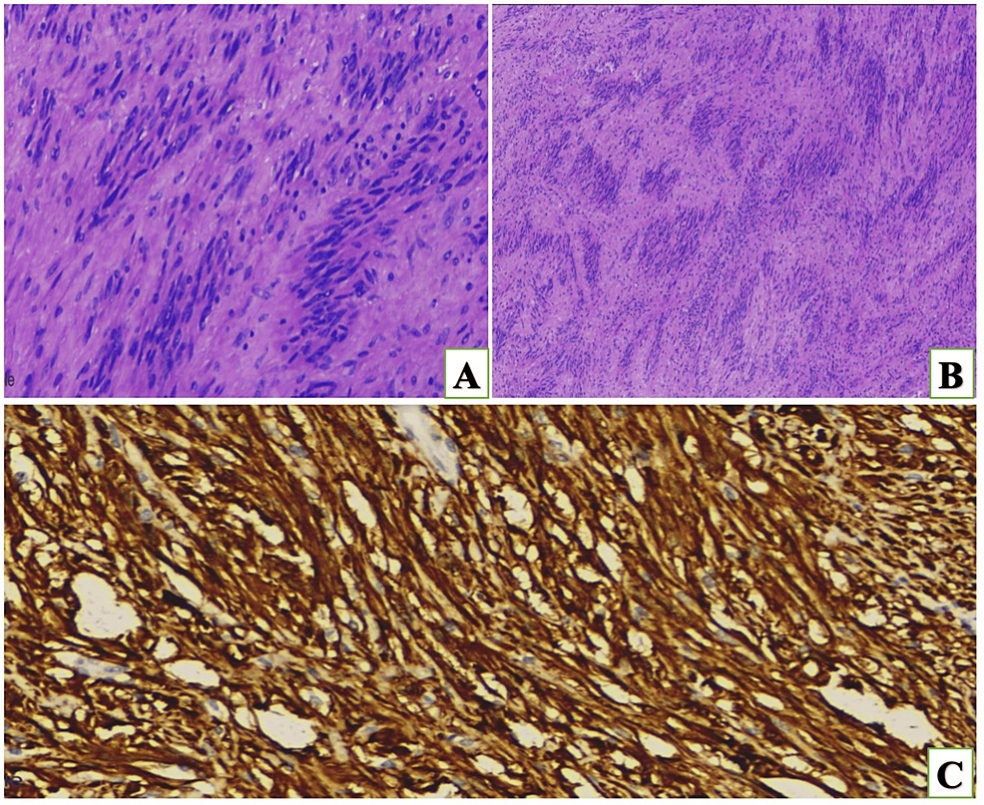
A) Histopathology image showing hypocellular and hypercellular areas; B) Antoni A and B areas; C) immunohistochemistry showing diffuse cytoplasmic positivity with s-100

On gross examination, a lobulated grey tan mass measuring 9.5×9×5 cm appears to arise from the lesser curvature of the stomach protruding into the lumen with the external surface covered by serosa and cut section shows a well-defined solid lesion with vague nodularity arising from submucosa without necrotic or hemorrhagic areas. On microscopic examination, a well-encapsulated lesion composed of spindle cells arranged in short fascicles and containing cellular areas with nuclear palisading (Antoni A) alternating with paracellular areas (Antoni B; Figure 2).

Peritumoral lymphoid aggregates and 1to2/5 mm2 mitotic activity are noted in the tumor tissue. Immunohistochemistry (IHC) shows the tumor stain positive for S100 (Figure 2C) glial fibrillary acidic protein (GFAP) and P16, whereas it was negative for CD117, DOG1, smooth muscle actin (SMA), and CD34. The final diagnosis of gastric schwannoma was made. The postop course was uneventful, and the patient was discharged on postoperative day five in a stable condition.

## Discussion

Gastrointestinal mesenchymal tumors are mainly composed of gastrointestinal stromal tumors (GIST), leiomyoma or leiomyosarcoma, and schwannomas, with GIST representing the largest group [5, 6]. They rarely occur in the GI tract and are thought to develop from the nerve plexus of the gut wall. The most common site of GI schwannoma is the stomach, followed by the colon and rectum, whereas small intestinal and esophageal schwannomas are extremely rare [3, 4].

Gastric schwannoma can occur at any age but is most commonly seen in the fifth and sixth decades, with female predominance [7]. They constitute 0.2% of all gastric tumors, and malignant conversion is extremely rare; only a few cases have been reported in the literature. They are commonly located in the middle third of the stomach along the lesser curvature, with the majority of tumors involving submucosa and muscular propria lined by intact mucosa without invading the adjacent structures. Gastric schwannomas are mostly asymptomatic; however, in a few cases, they can also present with nonspecific gastric symptoms, which include abdominal discomfort or pain, digestive symptoms, palpable mass if the tumor is large and exophytic and bleeding in case of deep ulceration [8].

The preoperative definitive diagnosis of gastric schwannoma is very difficult as there are no unique features seen on any diagnostic modalities. However, they are helpful in the characterization of the tumor and narrow the differential. On contrast-enhanced computed tomography, gastric schwannoma appears as a homogeneous, contrast-enhanced tumor, whereas GIST shows heterogeneous enhancement due to cystic degeneration. Cystic change, cavity formation, necrosis, or calcification are uncommon in gastric schwannomas [9]. On endoscopy, they appear grossly as elevated submucosal lesions, and a central ulcer may be seen in 25-50% due to ischemic changes in the overlying intact mucosa [10]. Considerable false negatives are seen with endoscopic biopsy diagnosis, so it is not as effective as expected in the diagnosis of gastric schwannomas. On magnetic resonance imaging (MRI), these tumors show low to intermediate signal intensity on T1-weighted images and high signal intensity on T2-weighted images. The best method to diagnose small lesions is endoscopic ultrasound (EUS), whereas transabdominal ultrasonography is for larger tumors [8].

The definitive diagnosis of gastric schwannomas requires pathological and immunohistochemical (IHC) examination. Histologically, gastric schwannoma shows focal atypical spindle cells that are arranged in a micro trabecular-microvascular pattern and a prominent lymphoid cuff surrounding the tumor, often with germinal centers. IHC is used to differentiate gastric schwannoma from other GI mesenchymal tumors; gastric schwannoma strongly and diffusely stains positive for S100, vimentin, and glial fibrillary acidic protein (GFA) and is consistently negative for CD117, DOG -1, CD 34 and smooth muscle actin (SMA). GIST shows positive stains for CD 117, DOG-1, and CD 34, whereas leiomyoma expresses CD34, desmin, and SMA [11, 12].

Surgical resection of the tumor should be considered as there is a need to establish a definitive diagnosis and to prevent possible complications such as bleeding or pyloric stenosis. Complete surgical resection, either through a classical or laparoscopic approach, offers curative treatment as well as treatment of choice for gastric schwannomas [13]. The size and location of the tumor will decide the extent of gastric resection to be performed. Local extirpation, wedge resection, partial, subtotal, or even total gastrectomy are all acceptable operations and show an excellent prognosis after resection. Many case reports do not report recurrence [8, 14, 15].

## Conclusions

Schwannoma is the most common benign nerve sheath tumor, rarely arising from the GI tract. It is difficult to differentiate on imaging from other mesenchymal tumors like GIST, which is only differentiated with Immunohistochemistry. Complete excision has a good prognosis compared to GIST, which has malignant potential.
